# Efficient use of binned data for imputing univariate time series data

**DOI:** 10.3389/fdata.2024.1422650

**Published:** 2024-08-21

**Authors:** Jay Darji, Nupur Biswas, Vijay Padul, Jaya Gill, Santosh Kesari, Shashaanka Ashili

**Affiliations:** ^1^Rhenix Lifesciences, Hyderabad, Telangana, India; ^2^CureScience, San Diego, CA, United States; ^3^Department of Translational Neurosciences, Pacific Neuroscience Institute and Saint John's Cancer Institute at Providence Saint John's Health Center, Santa Monica, CA, United States

**Keywords:** binned data, imputation, missing data, time series data, wearable

## Abstract

Time series data are recorded in various sectors, resulting in a large amount of data. However, the continuity of these data is often interrupted, resulting in periods of missing data. Several algorithms are used to impute the missing data, and the performance of these methods is widely varied. Apart from the choice of algorithm, the effective imputation depends on the nature of missing and available data. We conducted extensive studies using different types of time series data, specifically heart rate data and power consumption data. We generated the missing data for different time spans and imputed using different algorithms with binned data of different sizes. The performance was evaluated using the root mean square error (RMSE) metric. We observed a reduction in RMSE when using binned data compared to the entire dataset, particularly in the case of the expectation–maximization (EM) algorithm. We found that RMSE was reduced when using binned data for 1-, 5-, and 15-min missing data, with greater reduction observed for 15-min missing data. We also observed the effect of data fluctuation. We conclude that the usefulness of binned data depends precisely on the span of missing data, sampling frequency of the data, and fluctuation within data. Depending on the inherent characteristics, quality, and quantity of the missing and available data, binned data can impute a wide variety of data, including biological heart rate data derived from the Internet of Things (IoT) device smartwatch and non-biological data such as household power consumption data.

## 1 Introduction

Time series data are a continuous collection of observations of a single entity or multiple entities at different time points, which may or may not be equally spaced (Shumway and Stoffer, 2017). It is generated by various sources belonging to different domains, such as physiology, economics, environment, astronomy, business, and finance. Another important source of time series data is different types of Internet of Things (IoT) devices, including smart wearables that collect physiological data (Takiddeen and Zualkernan, 2019). In most cases, the seamless operations generate “big data” due to the continuous generation of data at a high sampling rate. However, observation may become unavailable at specific time points for various reasons, resulting in the generation of missing data. In the databases, these missing data are often stored as NULL values. This loss of information distorts the quality and properties of the remaining available data. It not only makes the downstream analysis challenging but also influences the inferences (Kreindler and Lumsden, 2016; Ahn et al., 2022). It also affects machine learning (ML) algorithms, which are frequently used for downstream analysis of time series data (Ngueilbaye et al., 2021). Hence, excluding the missing part is not a rational solution. Rather, the missing data must be synthetically generated or imputed using the available data from other time points. Imputation is also required when data are unfit for analysis due to noise and need to be replaced by the imputed data (Honaker and King, 2010).

There are multiple algorithms for imputing data (Pratama et al., 2017; Darji et al., 2023). ML-based methods are also being leveraged to impute missing data (Ngueilbaye et al., 2021; Alabadla et al., 2022). The performance of the algorithm is determined by the amount of missing data, types of missing data, kind of data, sampling rate of the data, amount of the data, fluctuation of the remaining data, and requirement of the downstream analysis. Overall, it depends on the innate content and nature of the data. Missing data can be of different types such as missing at random (MAR), missing completely at random (MCAR), and missing not at random (MNAR) (Junger and Ponce de Leon, 2015; Mir et al., 2022). Time series data are available of different types, which may or may not have seasonal features (Franses, 1991; Afrifa-Yamoah et al., 2020). The sampling rate of the data is another important feature that determines the number of data points missing in a given time span of missing data. It also determines the number of data points of available data needed to impute the missing data. The success of imputation is also dependent on the total amount of data and the fluctuation within the data. Imputation becomes challenging for highly fluctuating data as it deemphasizes the inherent trend within the data. The choice of the algorithm is also determined by the specific requirement of the downstream analysis of the imputed time series data. Moreover, proper employment of the available data is also crucial. In our earlier report, we have shown that the use of bins of data provides better imputation compared to the use of the entire data (Chakrabarti et al., 2023).

In this current report, we extend our studies on the utilization of binned data for missing data of different time spans of univariate time series data collected from different sources. We primarily used heart rate data derived from smartwatches worn by the healthy volunteers. We synthetically made the missing data for 1- and 5-min time spans and then imputed the data using different algorithms. The performance of imputation was quantified by using root mean square error (RMSE) as a metric. Through our analysis, we report on the circumstances that determine that binned data will provide better imputation compared to the imputation using the entire dataset. We report the span of missing data, sampling rate of the data are crucial factors. We also report the role of data fluctuation using missing data from different time periods of day depending on whether the volunteers remain physically active or inactive. We further extended our studies to non-physiological data. We investigated the effectiveness of binned data in another time series data of power consumption.

## 2 Materials and methods

### 2.1 Data acquisition

We acquired two types of time series data, namely, heart rate data which are biological data and power consumption data. The first one was acquired by ourselves, and the second was publicly available data.

#### 2.1.1 Heart rate data

Heart rate data was collected as a part of the clinical trial NCT05106725. This is an IRB-approved non-interventional study, and informed consent was collected from all subjects recruited in the trial. The data were extracted from the wearable IoT device Fitbit Inspire 2 worn by four healthy male volunteers. The volunteers wore their respective devices over the whole day. From a pool of 4 months' data, we selected 30 random days for each volunteer having no missing data. The heart rate data were available at 5-s intervals and converted to 1-min intervals after preprocessing.

#### 2.1.2 Power consumption data

The household electric power consumption database was downloaded from the UC Irvine Machine Learning (Hebrail and Berard, 2012). It is an energy consumption dataset for a house, containing over 2 million measurements recorded at an interval of 1 min from December 2006 to November 2010. From the pool of 47 months' data, we used 1 month's (March 2007) data to evaluate the efficiency of using binned data for imputation.

### 2.2 Missing data generation

We synthetically generated missing data by deleting data from days having complete data. Then, we imputed the missing data and compared the imputed data with the original data. We used data for two different time frames of a day, which we refer to as the “active period” and “inactive period.” The “inactive period” refers to 3–4 a.m. when our volunteers were asleep and hence inactive. The “active period” refers to 3–4 p.m. when our volunteers were active in their different daily activities. For 5-min missing period, we chose 3:22–3:27 a.m. and 3:22–3:27 p.m. For 1-min missing period, we chose 3:23–3:24 a.m. and 3:23–3:24 p.m. A similar time span was chosen for power consumption data too. Here also, during the “inactive period,” the power usage was stable compared to the “active period.”

### 2.3 Data binning

The data from different days were binned into different sizes around the missing data period. For 1-min missing data, bin sizes were 1-, 2-, 3-, 4-, 5-, 10-, 15-, 30-, and 45-min, and 1–6 h at an interval of 1 h. For 5-min missing data, we used bins with bin sizes starting from 5 min. In the case of power consumption data—while imputing 15-min missing data—we used bin sizes starting from 15 min.

### 2.4 Data imputation

As described in our previous report (Chakrabarti et al., 2023), we employed the expectation–maximization (EM) (Molenberghs and Verbeke, 2005), and random forest (RF) (Tang and Ishwaran, 2017) algorithms using Impyute and missForest methods, respectively, from missingpy Python module (Stekhoven and Bühlmann, 2012). Iterative imputation (II) (Templ et al., 2011), k-nearest neighbors (kNN) (Zhang, 2012), and SimpleImputer (SI) were employed using IterativeImputer, kNNImputer, and SimpleImputer, respectively, from scikit-learn Python module (Pedregosa et al., 2011). The training of all models was conducted using a heart rate dataset spanning over 30 days. We specifically focused on the data bins of different window sizes to fit the model. Subsequently, these trained models were employed to predict missing values, specifically spanning 1, 5, and 15 min. To evaluate the performance of imputation algorithms, we used RMSE as a metric (Khayati et al., 2020). RMSE calculates the disparity between the actual and imputed values, providing a quantitative measure of the accuracy of our models in handling missing data.

## 3 Results

### 3.1 Imputation of heart rate data

**Figure 1:**
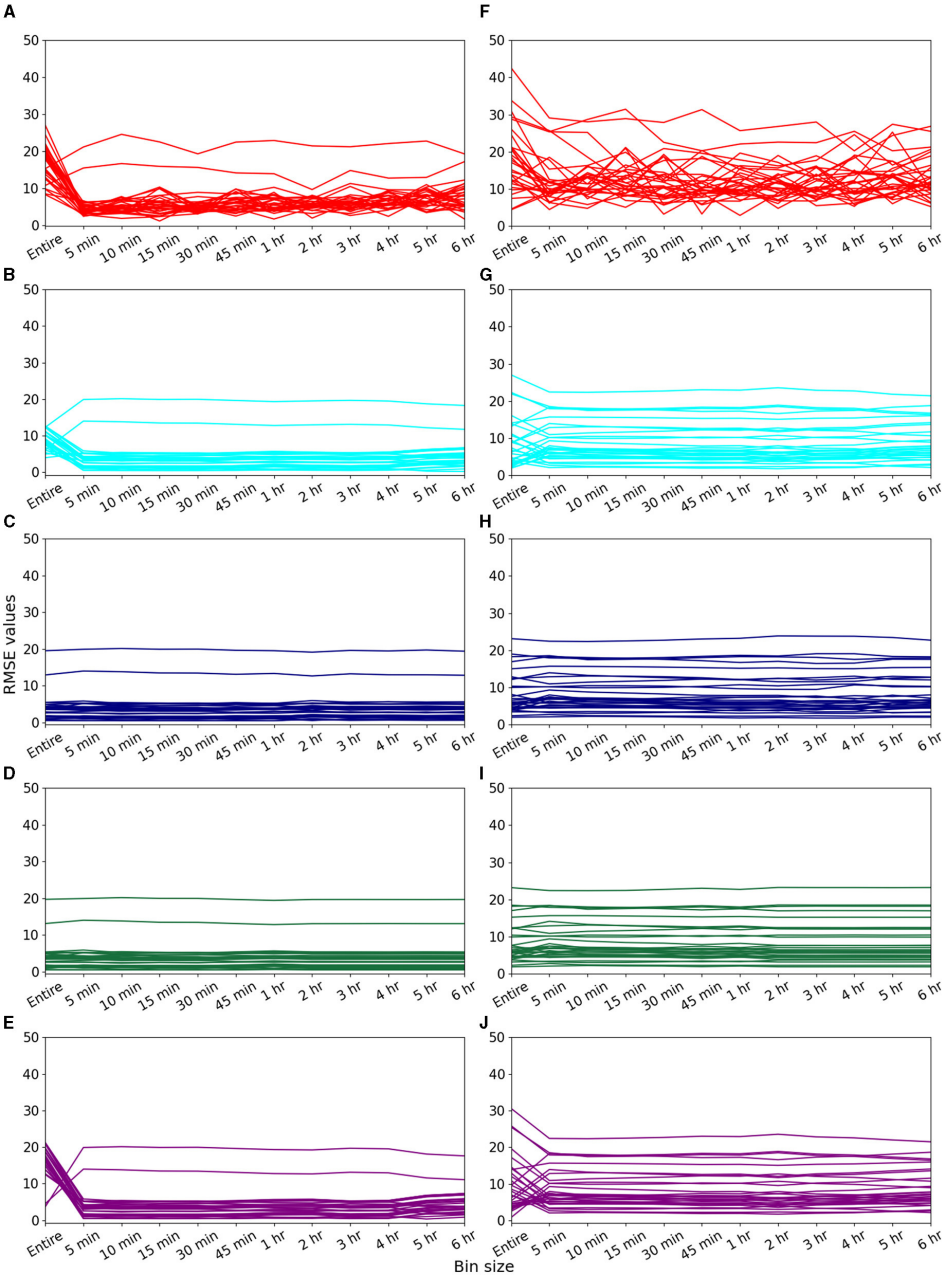
Imputation of 5 min of missing heart rate data for volunteer V1. Variations in RMSE when data of different bin sizes were used for imputing missing data of “inactive” period of 3:22–3:27 a.m. using **(A)** EM, **(B)** II, **(C)** kNN, **(D)** RF, and **(E)** SI methods. Variations in RMSE when data of different bin sizes were used for imputing missing data of “active” period of 3:22–3:27 p.m. using **(F)** EM, **(G)** II, **(H)** kNN, **(I)** RF, and **(J)** SI methods.

Figure 1 illustrates the variation of RMSE values for imputing 5-min missing data using data of different bin sizes and imputation methods. Each panel shows the data from 30 observed days and the data obtained from volunteer V1. We observed that the RMSE obtained using binned data for imputation is lower compared to the RMSE obtained when the entire data were used for imputation. Figures 1A–E shows results for missing data for 5 min from 3:22 to 3:27 a.m. during the “inactive period” of the volunteer. Figures 1F, J displays the result for missing data for 5 min from 3:22 to 3:27 p.m. during the “active period” of the volunteer.

Figure 1A reveals for the EM algorithm, RMSE values were reduced for 29 days when 1-h binned data was used. Likewise, in the active period, EM demonstrated improved imputation results for 24 days for 1-h binned data (Figure 1F), further underscoring the algorithm's proficiency across both periods. We further explored the performance of other algorithms. Compared to the entire dataset, for the II method, RMSE was reduced for 28 and 15 days for inactive and active periods, respectively, for 1-h binned data (Figures 1B, G). Similarly, for the SI method, RMSE was reduced for 28 days for the inactive period and 17 days for the active period when 1-h binned data were used (Figures 1E, J). However, for kNN and RF, we do not observe a reduction in RMSE when binned data was used for imputation for both inactive and active periods. We further imputed missing data for 1 min for both active and inactive periods of the same volunteer (Supplementary Figure S1).

### 3.2 Quantitative evaluation of imputation

**Figure 2:**
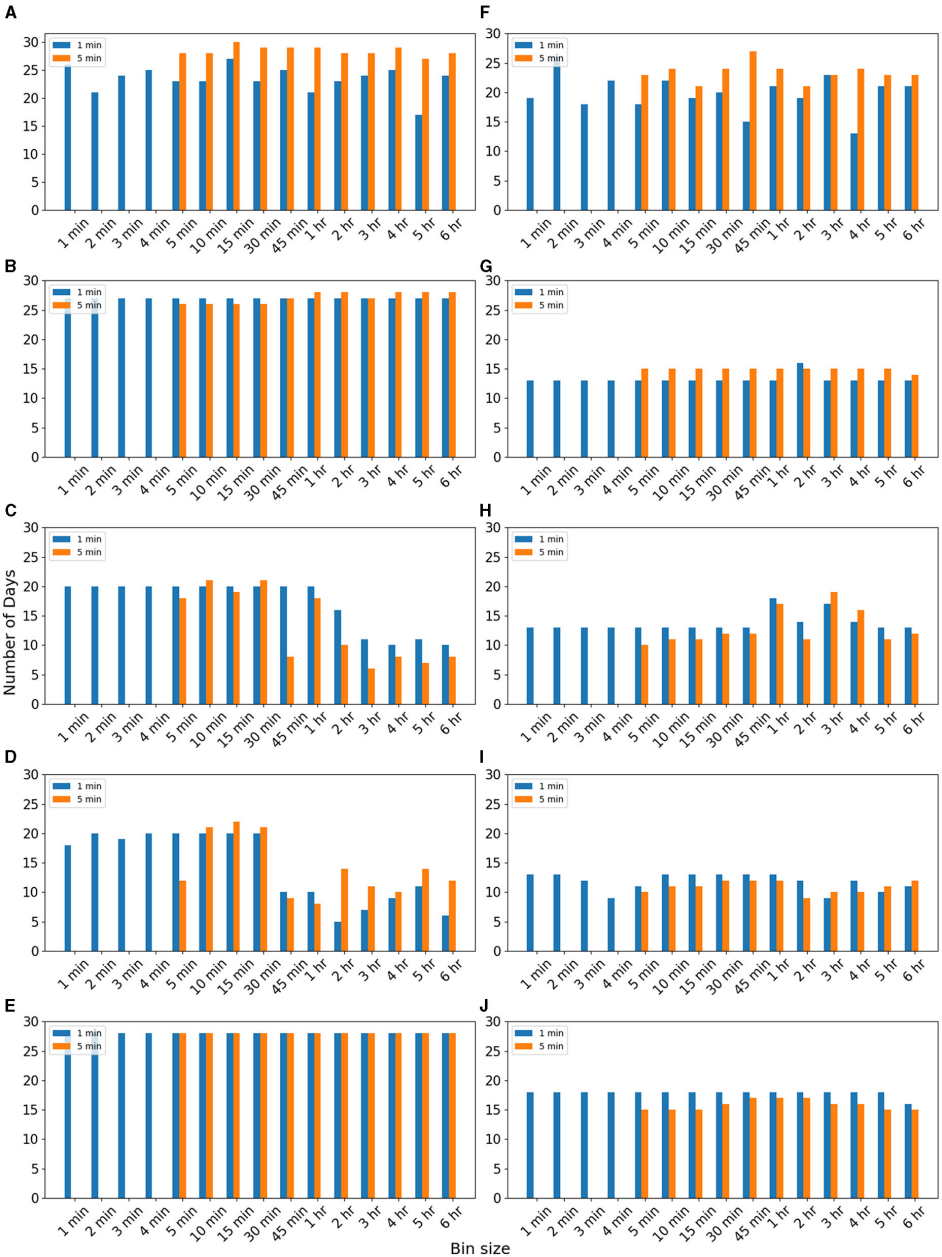
Number of days RMSE reduced when data were used for different bin sizes for imputing 5-min missing data of “inactive” period of using **(A)** EM, **(B)** II, **(C)** kNN, **(D)** RF, and **(E)** SI methods. Number of days RMSE was reduced when data were used for different bin sizes for imputing 5-min missing data of “active” period using **(F)** EM, **(G)** II, **(H)** kNN, **(I)** RF, and **(J)** SI methods. Data are for volunteer V1.

Figure 2 compares the performance of different bin-sized data for imputing 5- and 1-min missing data. We observed for EM, II, and SI algorithms, RMSE reduced when binned data were used (Supplementary Figure S1). Here also kNN and RF did not respond to binned data (Supplementary Figure S1). We observed for 1-min missing data of inactive period RMSE reduced for a maximum of 27 days when 15-min bin data were used for the EM algorithm (Figure 2A). In the case of 5-min missing data, RMSE reduced for a more significant number of days (30 days) when 15-min bin data were used for the EM algorithm (Figure 2). Figure 2A reveals the number of days for which RMSE reduced is more for imputing 5-min missing data for each bin size. It implies that the binned data are more effective for imputing 5-min missing data. A similar trend was observed for the active period also (Figure 2F). We observe the number of days RMSE reduced is more for 5-min missing data for all bin sizes.

However, for the II algorithm, binned data worked equally for both 5- and 1-min missing data from the inactive period (Figure 2B). However, for the active period, binned data was less effective for 1-min missing data (Figure 2G). It implies that II binned data work better for the inactive period. For kNN and RF algorithms, we observe that the bin sizes < 45 min are useful for 1- and 5-min missing data for inactive periods. We do not find any clear trend for the active period due to the lesser sensitivity of these algorithms. For the SI method also, binned data are effective for inactive periods. Similar observations were noted for the data from other volunteers (Supplementary Figures S2–S4). Supplementary Figure S5 shows the relative change in RMSE when binned data were used compared to the RMSE when the entire data were used for imputation. Supplementary Figure S6 shows the reduction in average RMSE when binned data are used. Overall, we observe that the binned data are effective for imputing 5-min missing data of inactive period. However, in either case, we do not find any optimal choice of bin size.

### 3.3 Imputation of power consumption data

**Figure 3:**
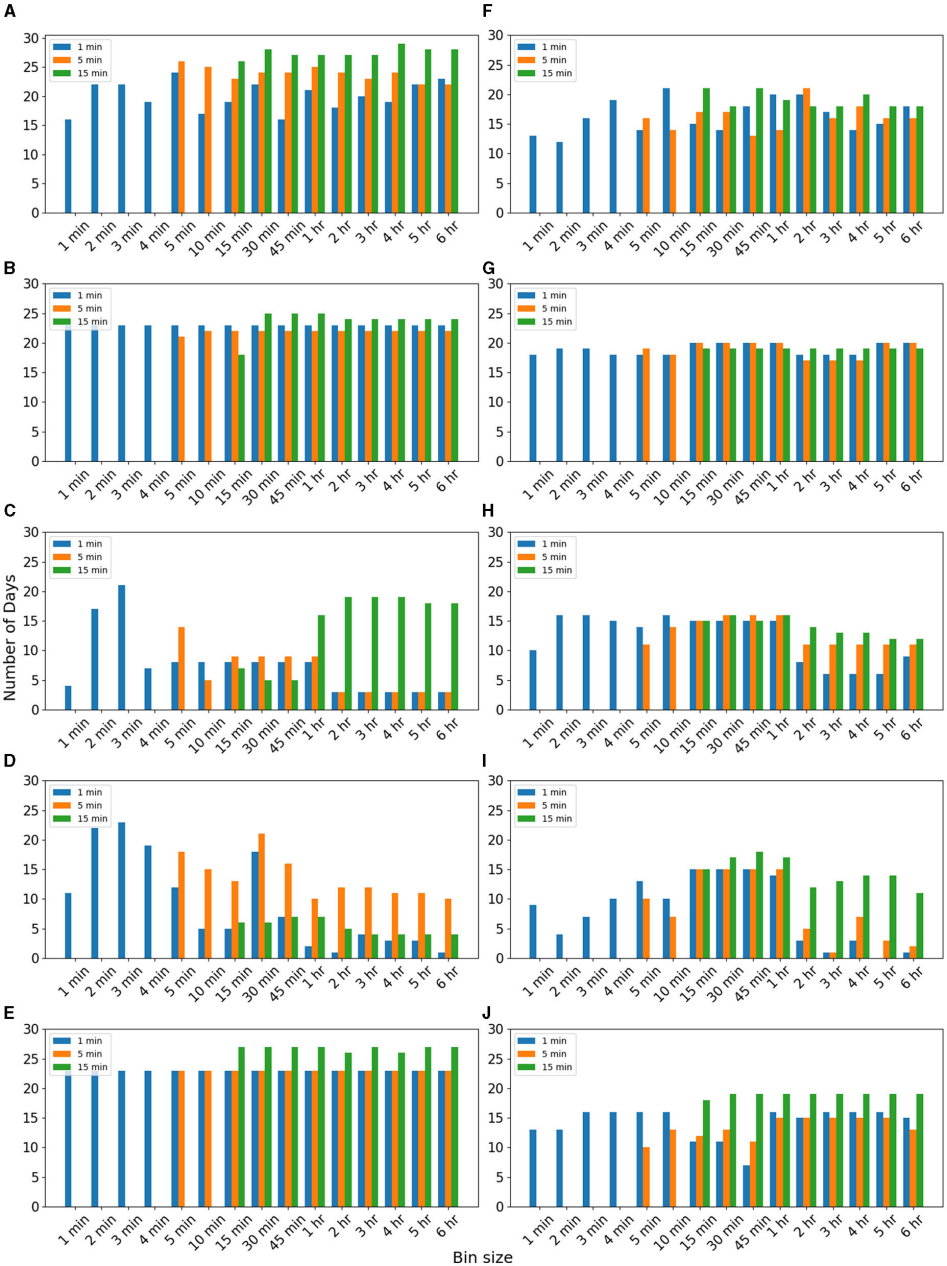
Imputation of power consumption data. Number of days RMSE was reduced when data were used for different bin sizes for imputing 5-min missing data of “active” period of using **(A)** EM, **(B)** II, **(C)** kNN, **(D)** RF, and **(E)** SI methods. Number of days RMSE was reduced when data were used for different bin sizes for imputing 5-min missing data of “inactive” period using **(F)** EM, **(G)** II, **(H)** kNN, **(I)** RF, and **(J)** SI methods.

To explore the effectiveness of binned data on the imputation of non-biological time series data, we analyzed power consumption data. Data details are mentioned in Section 2. Figure 3 compares the number of days RMSE reduced for imputing missing data for 1, 5, and 15 min for different algorithms. We observed for 1-min missing data of inactive period, RMSE reduced for a maximum of 25 days when 5-min bin data were used for the EM algorithm (Figure 3A). In the case of 5-min missing data, RMSE was reduced for more days (27 days) when 5-min bin data were used for the EM algorithm (Figure 3A). For 15-min missing data, RMSE reduced for a more significant number of days (29 days) when 4-h bin data were used for the EM algorithm (Figure 3A). It is consistent with our observation in the case of heart rate data (Figure 2A) although the bin sizes changed. We observe that for the active period, for 1-, 5-, and 15-min missing data, RMSE reduced when binned data were used. The reduction is more for 15-min missing data (Figure 3F).

## 4 Discussion

### 4.1 Role of time span

We earlier reported binned data provides better-imputed results for 15-min missing data with a defined optimal bin size (Chakrabarti et al., 2023). In the current study, we observed when heart rate was missing for 5 min, binned data still provided better imputation for the majority of the days. However, for 1-min missing data, binned data provided better imputation for a smaller number of days. A similar trend was observed while imputing power consumption data. It indicates that the time span of the missing data determines the usefulness of binned data for imputation. The reason may be due to the inherent pattern in the heart rate profile of a person. A person's lifestyle leaves a distinctive imprint on their heart rate (Honório et al., 2022). As lifestyle follows some daily repetitive activities, heart rate pattern also carries that profile. Hence, for imputing missing data of a particular period of any day, data from other days around the same time period are adequate. Also, the time span should be sufficient to carry the signature of the regularly repetitive work. Reduction of time span also implies reduction of data points. Hence, the sampling rate of the data is also crucial. This is the reason that the binned data are more effective for imputation of missing data of a higher time span of 15 min compared to the 5 min and even lower 1-min missing data. We conclude that the time span of missing data and sampling rate play a competitive role.

### 4.2 Role of active and inactive periods

**Figure 4:**
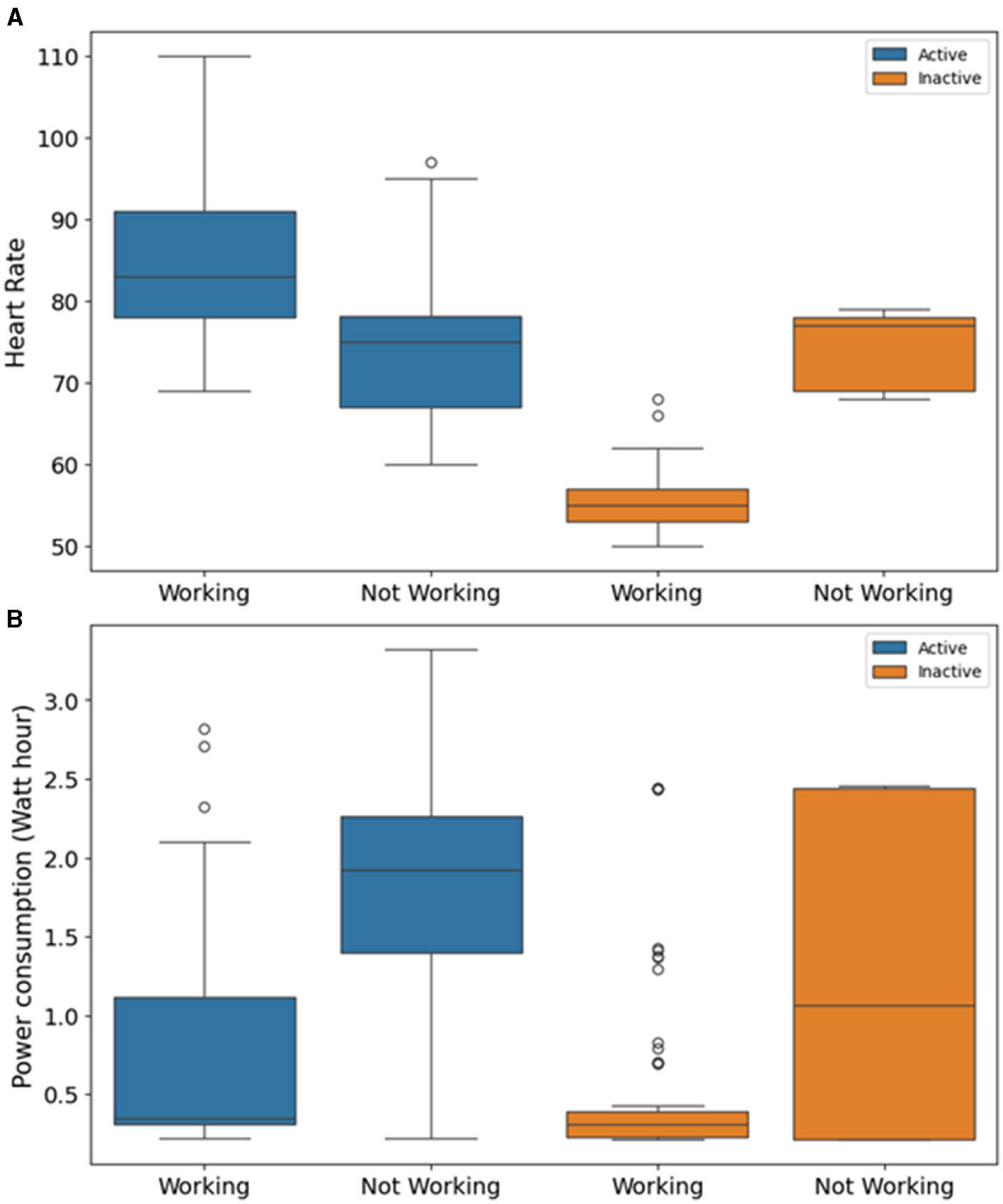
Box plot for fluctuation in 1 h binned data around 5-min missing data for the days when binned data worked and not worked using EM algorithm for **(A)** heart rate data and **(B)** power consumption data.

We also observed that binned data provides better-imputed data for more days for the inactive period of the day. The inactive period is characterized by lesser fluctuation in data. The higher fluctuating binned data is not suitable for imputation. Figure 4A shows fluctuation in heart rate data was quite low for the days binned data worked in inactive period. However, in the case of the active period, the fluctuation was comparably high for both binned data working and non-working days. It is reflected in the fact that the number of days RMSE was reduced was less for the active period compared to the inactive period (Figures 2A, F). In the case of power consumption data, for both the inactive and active periods, the fluctuation was high when binned data did not work for 5-min missing data (Figure 4B).

### 4.3 Choice of algorithm

In concordance with our earlier observations, among different algorithms, EM still appears to be the most effective. The current study uses data where missing data are of MAR type. The EM method does not have any hyperparameters and uses missing values as hidden variables. It makes EM effective in the case of imputing MAR-type data (Theodoridis, 2020). Among the other methods, RF did not work well in consistent with earlier observations in the case of outcome-dependent MAR data (Hong and Lynn, 2020).

### 4.4 Advantages and limitations

The use of binned data implies the use of data from a specific span. In other words, it uses censored time span data (Støvring and Kristiansen, 2011). It is advantageous as it uses less amount of data, hence making faster imputation of real-time data possible. As it considers data from a specified span, the presence of noise and outliers beyond that span does not affect the imputation process. The binning method is also applicable to a wide variety of imputation algorithms.

However, the major challenge of using binned data for imputing missing data occurs when data of missing periods deviate from the usual characteristics of the available data of similar time periods and the span of other days. The activity of a person is reflected in his or her heart rate data. If a person deviates from their routine activity for a particular day and if that data are missing, in that case, the use of binned data may not be useful for imputation. The technical challenge of using binned data includes generating binned data of optimal bin size. Since time series data often carry personalized features, the choice of bin size also needs to be personalized. The so-called “active” and “inactive” periods may vary for different data sources, even for similar data types.

## 5 Conclusion

We observed that using binned data provides better-imputed data for different types of time series data. The advantage of the use of binned data includes the requirement of a lesser amount of data along with faster computation. It can be integrated into big data analytics to fill the missing part. It will help in the faster functioning of automated devices such as IoT devices. Our extensive study shows that the usefulness of binned data is determined by the innate properties of the data, which include the span of the missing period, the sampling rate of the data, and the fluctuation within the available data. Furthermore, the optimal bin size could not be defined because of the lesser effectiveness of binned data when the sampling rate is comparable to the missing data time span. Overall, the current study will be helpful for time series data management across wide domains.

## Data Availability

The raw data supporting the conclusions of this article will be made available by the authors, without undue reservation.
