# Supplementary material for: Efficient use of binned data for imputing univariate time series data
Source: Front Big Data. 2024 Aug 21;7:1422650. doi: 10.3389/fdata.2024.1422650 (PMC11371617; doi:10.3389/fdata.2024.1422650)
Supplement: Supplementary file 1 [file Data_Sheet_1.docx]

**Supplementary materials**

**Efficient use of binned data for imputing uni-variate time-series data**


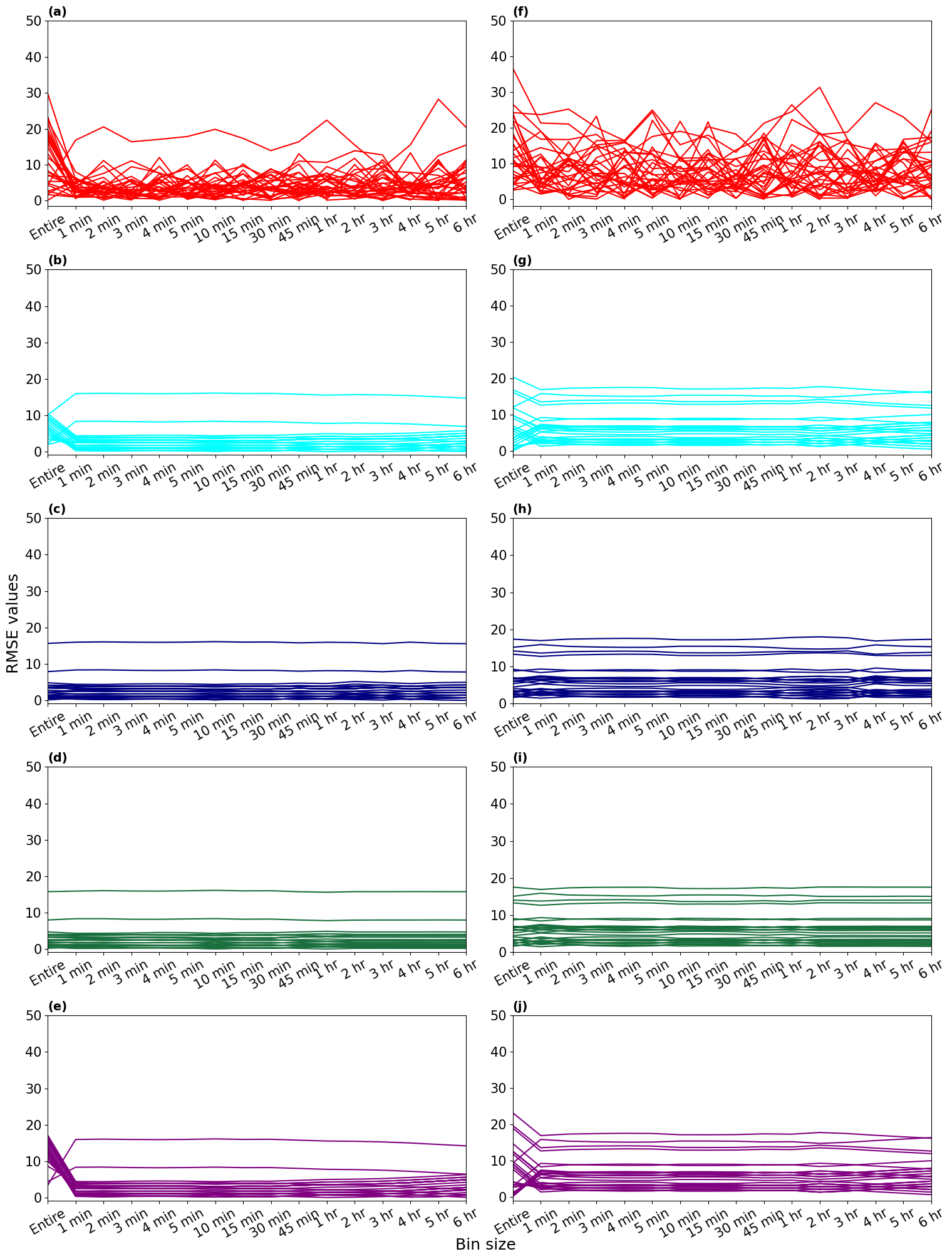


# Figure S1: Imputation of 1 min of missing heart rate data for volunteer V1. Variations in RMSE when data of different bin sizes were used for imputing missing data of ‘inactive’ period of 3:23 -3:24 am using (a) EM, (b) II, (c) kNN, (d) RF, and (e) SI methods. Variations in RMSE when data of different bin sizes were used for imputing missing data of ‘active’ period of 3:23 – 3:24 pm using (f) EM, (g) II, (h) kNN, (i) RF, and (j) SI methods.


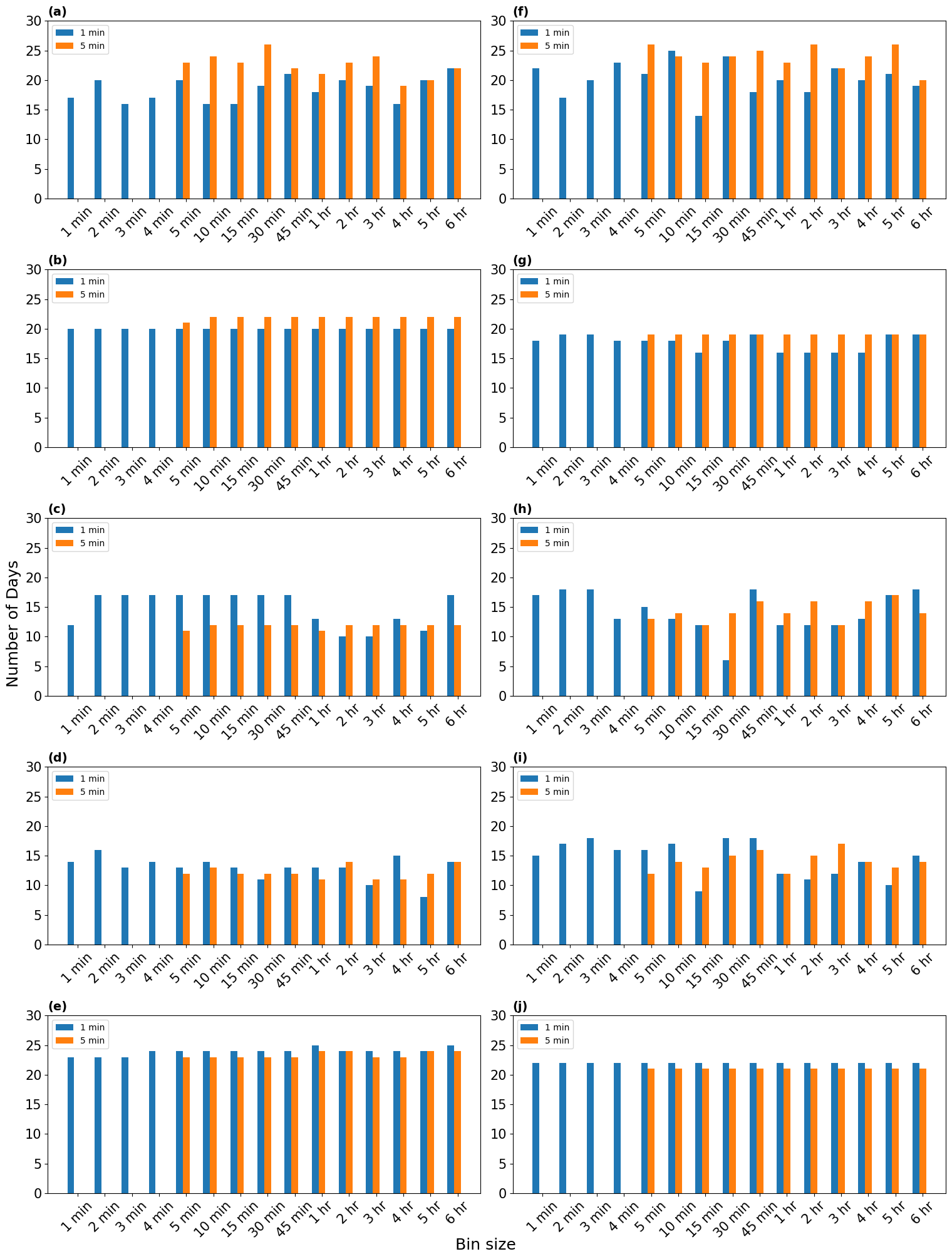


# Figure S2: Number of days RMSE reduced when data was used for different bin sizes for imputing 5 min missing data of ‘inactive’ period of using (a) EM, (b) II, (c) kNN, (d) RF, and (e) SI methods. Number of days RMSE was reduced when data was used for different bin sizes for imputing 5 min missing data of ‘active’ period using (f) EM, (g) II, (h) kNN, (i) RF, and (j) SI methods. Data is for volunteer V2.


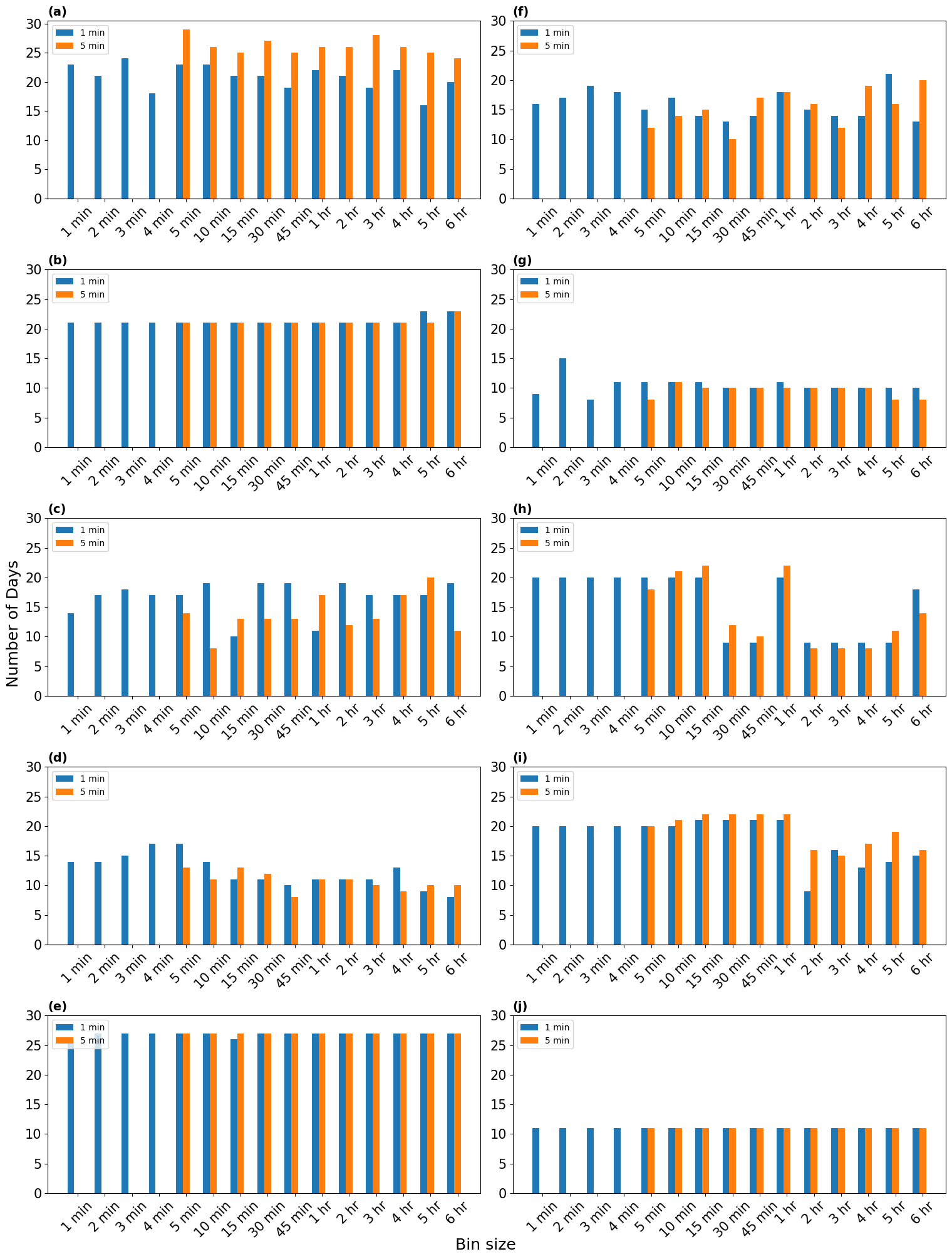


# Figure S3: Number of days RMSE reduced when data was used for different bin sizes for imputing 5 min missing data of ‘inactive’ period of using (a) EM, (b) II, (c) kNN, (d) RF, and (e) SI methods. Number of days RMSE was reduced when data was used for different bin sizes for imputing 5 min missing data of ‘active’ period using (f) EM, (g) II, (h) kNN, (i) RF, and (j) SI methods. Data is for volunteer V3.


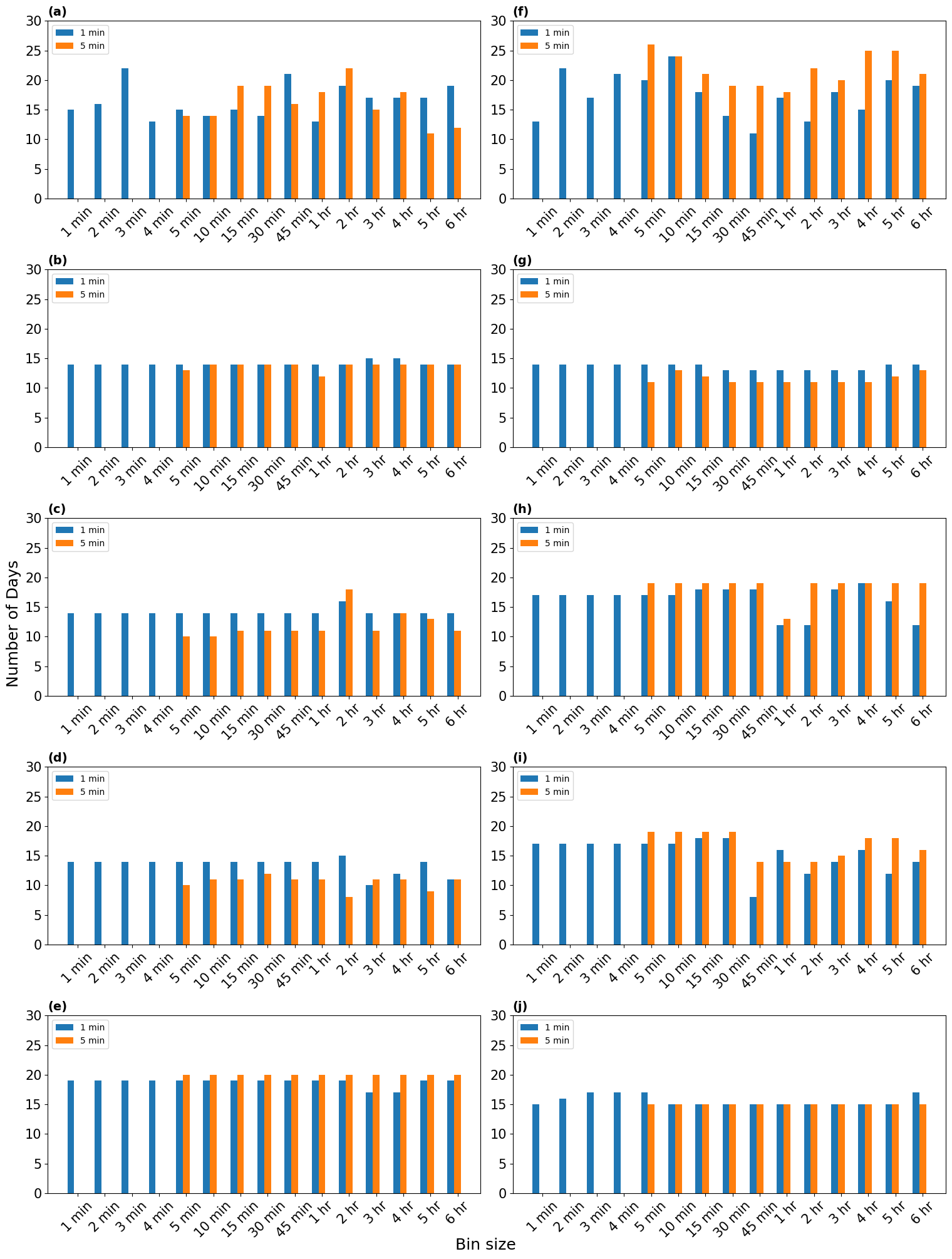


# Figure S4: Number of days RMSE reduced when data was used for different bin sizes for imputing 5 min missing data of ‘inactive’ period of using (a) EM, (b) II, (c) kNN, (d) RF, and (e) SI methods. Number of days RMSE was reduced when data was used for different bin sizes for imputing 5 min missing data of ‘active’ period using (f) EM, (g) II, (h) kNN, (i) RF, and (j) SI methods. Data is for volunteer V4.

**(a)**

| Bin Size | EM | II | KNN | RF | SI |
| --- | --- | --- | --- | --- | --- |
| Entire | 30.94 | 13.83 | 10.27 | 10.37 | 17.2 |
| 5min | 12.47 | 10.1 | 10.1 | 10.12 | 10.1 |
| 10min | 11.3 | 9.86 | 9.86 | 9.88 | 9.86 |
| 15mins | 12.62 | 9.89 | 9.89 | 9.86 | 9.89 |
| 30mins | 18.63 | 9.98 | 9.98 | 10.03 | 9.98 |
| 45mins | 11.45 | 10.23 | 10.23 | 10.24 | 10.23 |
| 1hr | 19.61 | 10.14 | 10.67 | 10.03 | 10.15 |
| 2hr | 17.09 | 10.69 | 10.36 | 10.38 | 10.69 |
| 3hr | 12.51 | 10.1 | 10.72 | 10.36 | 10.07 |
| 4hr | 13.7 | 9.95 | 10.87 | 10.4 | 9.84 |
| 5hr | 17.45 | 9.2 | 10.12 | 10.36 | 9.36 |
| 6hr | 9.6 | 8.85 | 10.1 | 10.39 | 8.93 |

| Bin Size | EM | II | KNN | RF | SI |
| --- | --- | --- | --- | --- | --- |
| Entire | 0 | 0 | 0 | 0 | 0 |
| 5min | 59.7 | 26.97 | 1.66 | 2.41 | 41.28 |
| 10min | 63.48 | 28.71 | 3.99 | 4.73 | 42.67 |
| 15mins | 59.21 | 28.49 | 3.7 | 4.92 | 42.5 |
| 30mins | 39.79 | 27.84 | 2.82 | 3.28 | 41.98 |
| 45mins | 62.99 | 26.03 | 0.39 | 1.25 | 40.52 |
| 1hr | 36.62 | 26.68 | -3.89 | 3.28 | 40.99 |
| 2hr | 44.76 | 22.7 | -0.88 | -0.1 | 37.85 |
| 3hr | 59.57 | 26.97 | -4.38 | 0.1 | 41.45 |
| 4hr | 55.72 | 28.05 | -5.84 | -0.29 | 42.79 |
| 5hr | 43.6 | 33.48 | 1.46 | 0.1 | 45.58 |
| 6hr | 68.97 | 36.01 | 1.66 | -0.19 | 48.08 |

**(b)**

# Figure S5: Statistical analysis of imputation for a given day when 5 min heart rate data was missing in ‘active period’. (a) The RMSE values obtained from different algorithms using binned data. (b) The change in RMSE in percentage compared to the respective ‘Entire’ data.

(a) (b)


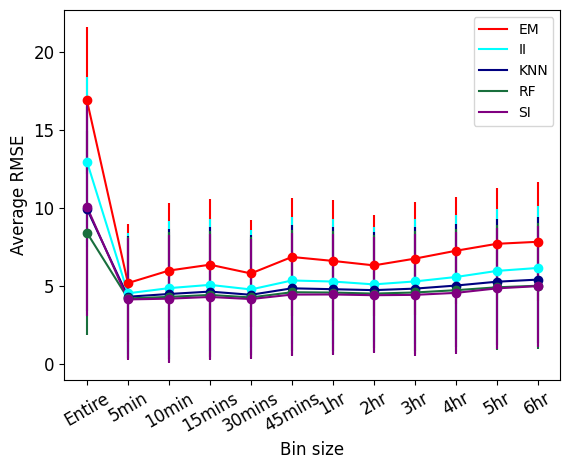

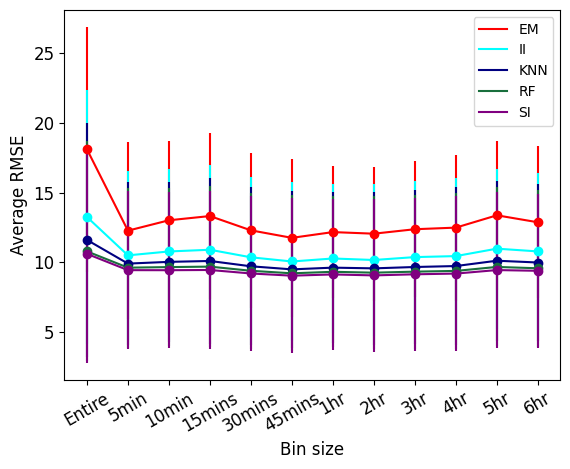


Figure S6: Variation of average values of RMSE for (a) inactive and (b) active periods with bin size. The average values were calculated from data of 30 days shown in Fig 1. The error bars represent standard deviations.
